# Automated splitting into batches for observational biomedical studies with sequential processing

**DOI:** 10.1093/biostatistics/kxac014

**Published:** 2022-05-10

**Authors:** Bram Burger, Marc Vaudel, Harald Barsnes

**Affiliations:** Computational Biology Unit (CBU), Department of Informatics, University of Bergen, 5008 Bergen, Norway, Proteomics Unit (PROBE), Department of Biomedicine, University of Bergen, 5020 Bergen, Norway, and Department of Medical Genetics, Haukeland University Hospital, 5021 Bergen, Norway; Department of Clinical Science, University of Bergen, 5020 Bergen, Norway; Computational Biology Unit (CBU), Department of Informatics, University of Bergen, 5008 Bergen, Norway and Proteomics Unit (PROBE), Department of Biomedicine, University of Bergen, 5020 Bergen, Norway

**Keywords:** Batch generation, Experimental design, Heuristic algorithm

## Abstract

Experimental design usually focuses on the setting where treatments and/or other aspects of interest can be manipulated. However, in observational biomedical studies with sequential processing, the set of available samples is often fixed, and the problem is thus rather the ordering and allocation of samples to batches such that comparisons between different treatments can be made with similar precision. In certain situations, this allocation can be done by hand, but this rapidly becomes impractical with more challenging cohort setups. Here, we present a fast and intuitive algorithm to generate balanced allocations of samples to batches for any single-variable model where the treatment variable is nominal. This greatly simplifies the grouping of samples into batches, makes the process reproducible, and provides a marked improvement over completely random allocations. The general challenges of allocation and why good solutions can be hard to find are also discussed, as well as potential extensions to multivariable settings.

## 1. Background

**Fig. 1.**
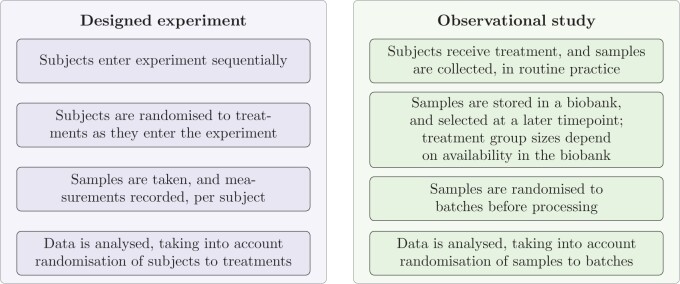
Examples of a designed experiment and an observational study, highlighting key conceptual differences and settings related to subject and sample randomization.

A common aim of experimental design is to find an appropriate configuration of runs to study a particular situation, for example, treatment vs. placebo (Rosenberger and Lachin, 2016; Bailey, 2008). For example in clinical trials, subjects enter an experiment sequentially and are randomized to treatments. Major concerns in such settings are treatment balance (e.g., a certain ratio of active treatment vs. placebo), selection bias (assignment to a particular treatment is not based on subject characteristics), and ascertainment bias (the assessment of the outcome can be biased if the treatment is known) (Rosenberger and Lachin, 2016). However, in observational biomedical studies, it is not uncommon that sample collection and sample processing are distinct parts of the study (Figure 1). For example, samples can be collected from subjects during routine practice, possibly as part of a biobanking effort, in which case treatment or disease status is part of the general subject characteristics, instead of being assigned to subjects in the course of the experiment (Krokstad *and others*, 2012; Rønningen *and others*, 2006; Sudlow *and others*, 2015). Such biobank-scale studies provide the opportunity to analyze differences and commonalities between related diseases, for example, across a spectrum of neurodegenerative diseases, and obtain a level of information that would most likely be impossible to achieve in a single study.

In these types of studies, the samples are often processed using omics technologies, which have their own distinct limitations concerning sample preparation and processing. For example, in proteomics and metabolomics using mass spectrometry the sample processing order can have a distinct influence on the measurements (Surowiec *and others*, 2018; Mertens, 2017). Similarly, sample preparation in the lab is labor intensive, with subsets of the samples potentially being processed by different people or spread across multiple days. Multiplexing samples, i.e., processing several samples together using chemical tags to subsequently deconvolve which molecules came from which sample, is currently limited to 18 samples (Li *and others*, 2021), and also has additional design challenges (Burger *and others*, 2021; Zhang *and others*, 2020).

Thus, the need often arises to split a study into smaller parts, which means having to decide which samples to process together. These distinct sets of samples are referred to as batches, and a key challenge is how to best design such batches for a given study where the set of available samples is fixed. Hence, treatment balance, selection bias, and ascertainment bias are still major concerns in this setting, but now with a focus on randomizing the collected samples to processing batches.

When a batch contains multiple sets of samples from each treatment regime, multiple equal parts of the batch, referred to as blocks, can separately be randomized using block randomization (Bailey, 2008; Box *and others*, 2005). However, the batches cannot always be divided into equal blocks, for example, when there are samples from five treatment regimes, while only eight samples can be processed at the same time. Hence, batches can, but do not necessarily, contain blocks.

The setting under consideration here is therefore different from what is generally assumed in the experimental design literature, where treatments can be assigned to subjects/samples, or settings can be changed in subsequent experimental runs (Bailey, 2008; Box *and others*, 2005). In such settings, the challenge is to find an allocation of treatments to possible subjects, while in our case the subjects and their treatments are already fixed before sample collection. Common to both settings is the need to randomize to avoid systematic bias, selection bias, accidental bias, and cheating by the experimenter (Bailey, 2008).

Thus, the sample processing order, and, if necessary, the creation of batches, are key when designing these types of studies. Furthermore, it is essential to avoid the introduction of confounders that bias the interpretation (Bailey, 2008; Box *and others*, 2005; Burger *and others*, 2021). While this can be achieved by domain experts, such ad hoc sample allocation is vulnerable to experimenter bias and does not scale to large numbers of samples, for example, for biobank-scale analyses. In such situations, an automated procedure is therefore required to quickly generate an allocation where all treatments and treatment contrasts can be estimated with similar precision. This will from here on be referred to as an optimal allocation, even though what is optimal for a given study can deviate from the setting under consideration here.

The available software tools for assigning subjects to blocks generally do so in the context of assigning treatment to subjects sequentially entering an experiment. In practice, for observational biomedical studies, the set of available samples is however often constrained or fixed, leaving the experimentalist with no choice but assigning them ad hoc. One notable exception, mainly in the context of genomics and transcriptomics, is Yan *and others* (2012), presenting a tool for block randomization for 2D settings. Here, we provide guidance for the 1D setting and analyze the specific challenges encountered.

Given the cohort and the maximum batch size of a study, the batch composition and sample order should (i) avoid unintended confounding and (ii) yield interpretable results even if something does not go as planned, for example, if (part of) a batch has to be discarded. In other words, each batch should present the highest diversity of treatments and the distribution of treatments should be as homogeneous as possible among the different batches. These are the same considerations as for generating blocks in block randomization, but it will often not be possible to have the same distribution of treatments in each batch. Similarly, while in this step we only consider the allocation of treatments to batches, the next step would be to use a process similar to stratified randomization to assign subjects/samples to fill the relevant places in the batches (Bailey, 2008; Box *and others*, 2005; Burger *and others*, 2021).

In this particular setting, the ideal situation is therefore when each treatment can be equally distributed across all of the batches, i.e., each batch contains ${N_i}/{B}$ subjects, with $i:1,\ldots,T$, where $T$ is the number of treatments, $N_i$ the number of subjects in treatment $i$, and $B$ the number of batches. For example, if there are 96 samples from five treatment regimes and we can only process eight samples at a time, for example, when using 8-well plates, each treatment regime is represented by $x \times 12$ samples, as each plate can then contain the exact same combination of treatment regimes. However, this is only possible if, for all $i$, $B$ divides $N_i$. If this is not the case, not all batches will be the same and it can become challenging to distribute the samples manually.

A natural simplification of the allocation problem follows directly from the above: if $B$ divides $N_i$, ${N_i}/{B}$ subjects receiving treatment $i$ are allocated to each batch; however, if $B$ is larger than $N_i$, there are more batches than subjects receiving treatment $i$. In order to have the batches as similar as possible, the subjects receiving treatment $i$ should all be allocated to different batches, i.e., each batch should contain at most one subject receiving treatment $i$.

On the other hand, if the number of subjects receiving treatment $i$$\left(N_i\right)$ is larger than the number of blocks $\left(B\right)$, at least one subject receiving treatment $i$ can and should be allocated to every batch. In fact, this is a combination of the two previous situations where the quotient of ${N_i}/{B}$ subjects receiving treatment $i$ is allocated to each batch, and the remainder of ${N_i}/{B}$ batches contain one more subject receiving treatment $i$.

The above simplification splits the allocation problem into two parts. First, for each treatment $i$, the quotient of ${N_i}/{B}$ subjects having received treatment $i$ is allocated to each batch. In the following, we will refer to this step as the *preallocation*. Second, allocate the remainders of ${N_i}/{B}$ subjects for each treatment $i$. The remainders are by definition all less than the number of batches, thus, the remaining problem is one of finding an appropriate design given the samples and batch sizes. As the allocation in the *preallocation* step is identical for all batches, it has no influence on the performance of the subsequent allocation for the remaining subjects.

Note that we use “allocate subjects having received treatment $i$” as a shorthand. All subjects having received treatment $i$ are assumed exchangeable, and thus we are really only allocating “a spot in a batch reserved for a subject having received treatment $i$.” The actual allocation of subjects can be done in a later step using a stratified randomization algorithm (see e.g., Rosenberger and Lachin, 2016).

This design is binary because all treatments occur at most once in each batch, and the blocks are incomplete in the sense that not all treatments can occur in a single batch (Bailey, 2004); as in this case all batches consist of a subset of the treatments, this can be seen as a binary incomplete-block design. If all treatments have the same number of subjects left to allocate, a balanced incomplete-block design with a known allocation strategy may exist (see Colbourn and Dinitz (2007) for an overview). Otherwise, trial and error may be required to allocate the treatments in such a way that every treatment shares a batch with every other treatment in proportion to their sample size. Each batch then becomes its own small version of the main study.

If each batch contains the same number of samples, each treatment occurs an equal number of times in the entire study, and the number of times two treatments occur together in a batch is the same for all pairs of treatments, this is called a balanced block design (Bailey, 2008). Whether each treatment occurs an equal number of times depends on many factors. Here, we assume that this is not necessarily the case and that the cohort, the set of samples included in the study, is fixed at the time of allocation. Additionally, whether each batch contains the same number of samples is dependent on the maximum batch size and the total number of subjects in the study.

An allocation that makes it possible to estimate one treatment variable with the highest precision, for example, an administered drug, may force an undesired allocation for another, for example, treatment duration. When the analytical model contains multiple variables, several competing aspects of the allocation thus have to be optimized, which leads to further complications. Similarly, when the treatment variables can take continuous values, this would be equivalent to subjects having unique values, using these variables as-is would mean that every subject could be considered unique and that all possible allocations would have to be considered. Categorization is commonplace (if only for the purpose of blocking) but has its drawbacks due to the introduction of fixed (possibly arbitrary) cut-points.

From a mathematical point of view, the main difficulty in assigning subjects to batches is that it is not possible to have fractional assignments of samples. The allocation problem thus becomes an integer programming problem, i.e., $x$ subjects of treatment $i$ are to be allocated to batch $k$, where $x$ is an integer. As one can only assign one or zero subjects of a treatment to each batch, with constraints on batch sizes and sample sizes, all options may have to be considered to find a solution that makes it possible to estimate the desired treatment contrasts with the highest possible precision given the study constraints. The search space for this problem thus grows exponentially as a function of the number of treatments and batches, i.e., this problem is NP-complete (Karp, 1972).

Optimal allocations are known for specific balanced settings (e.g., Bailey, 2004). However, when the cohort and batch sizes are not compatible with a standard setting the only option is to evaluate all possible allocations. Even though the search space can be limited to a binary allocation problem, for the single-variable setting with a nominal treatment variable (i.e., when one has Treatment A, B, C, $\ldots$), the number of options would still be $\prod{T \choose {b_k}}$, with $T$ the number of treatment levels and $b_k$ the number of samples in batch $k$. This quickly grows too large to calculate within a reasonable timeframe. By using the number of pairwise comparisons between all pairs of treatments as the guide for batch allocations, the number of possible solutions can be reduced to $\sum{T \choose {b_k}}$.

To address this challenge, we have implemented a heuristic algorithm to iteratively allocate a fixed cohort to batches based on a single nominal treatment variable. A heuristic cannot guarantee that an optimal allocation will be found, nor that it will produce better results than a random algorithm in every situation. Thus, we propose an approach where, in cases where manual allocation is challenging and calculating an optimal allocation is not feasible, our heuristic algorithm is run multiple times and the best of the generated allocations is selected.

## 2. Methods

### 2.1. Notation

In the following, we denote the total number of batches by $B$, the vector of batch sizes by $\boldsymbol{b}$, and the index of a specific batch by $k = 1,\ldots,B$. Similarly, the total number of treatments is denoted by $T$, with $\boldsymbol{t}$ the vector of sample sizes for each treatment $i$, and $i = 1,\ldots,T$, the index of a specific treatment. In addition, several standard matrices for different aspects of the allocation are used as detailed in Bailey (2004): (i) the incidence matrix $\boldsymbol{N}_{B \times T}$, with the treatments along with the columns and batches along the rows, indicates for each treatment the number of times it occurs in each batch, (ii) the concurrence matrix $\boldsymbol{\Lambda}_{T \times T}$, with treatments along the rows and columns, indicates for each pair of treatments how often they occur together in a batch. The diagonal of $\boldsymbol{\Lambda}$ is $N_i$, the number of subjects receiving treatment $i$. When each treatment occurs at most once in each batch, a so-called binary block design, the concurrence matrix $\boldsymbol{\Lambda} = \boldsymbol{N}^T\boldsymbol{N}$.

### 2.2. Complexity reduction

If the complete cohort is allocated to batches in one step, the number of options to consider would be in the order of $\prod{T \choose {b_k}}$. Conversely, when allocating one batch at a time, and at every step allocating a set of treatments together, the number of possible solutions to search decreases dramatically to $\sum{T \choose {b_k}}$. Note that this however comes at the cost of possibly missing the optimal allocation for the complete cohort, which will be detailed in the discussion. The number of calculations per batch can further be reduced to the order of $T \choose 2$ by instead allocating one subject at a time. The optimal set of treatments to allocate to a batch, given the previously allocated batches, but ignoring the subjects still to allocate, is in this context the set of treatments with the fewest pairwise comparisons between them.

### 2.3. Heuristic algorithm for sample allocation

**Fig. 2.**
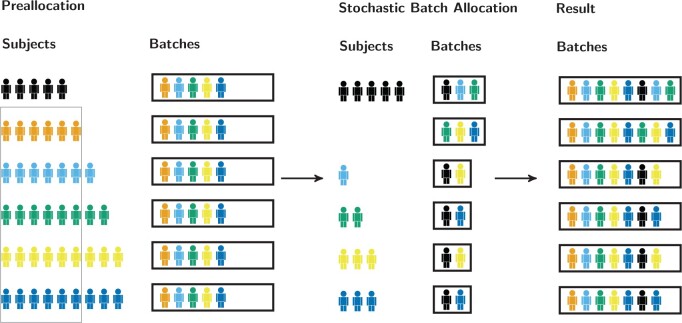
Overview of the three main steps of the proposed heuristic algorithm. Subjects receive one of six treatments (indicated by color). The vector $\boldsymbol{t}$ containing the number of subjects per treatment is [5, 6, 7, 8, 9, 9]. The vector $\boldsymbol{b}$ containing the batch sizes is [8, 8, 7, 7, 7, 7]. The preallocation step is part of the initialization of the algorithm, the SBA shows the part of the allocation problem our heuristic algorithm finds a solution for. Combining the two steps leads to a proposed batch allocation.

**Fig. 3.**
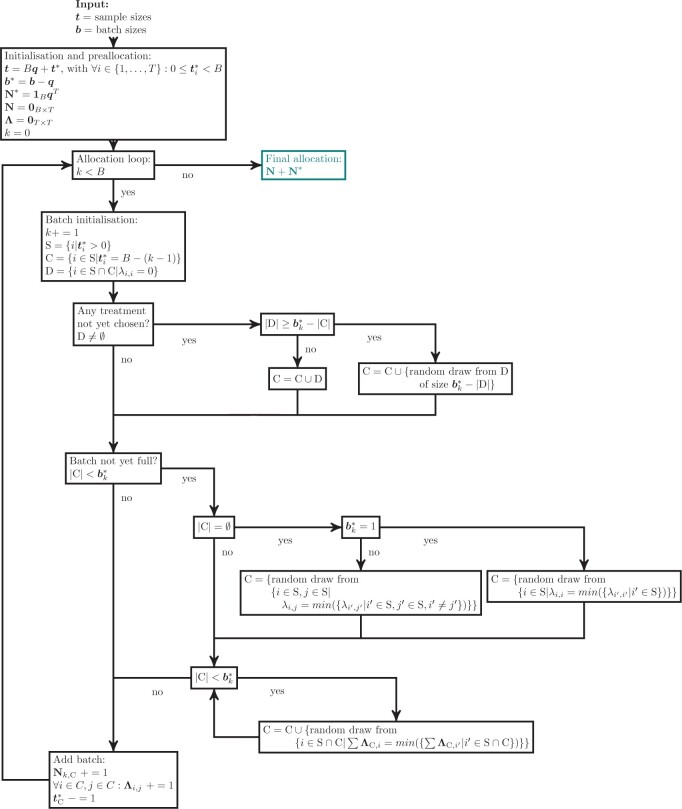
Overview of the proposed heuristic algorithm. The initialization step includes the *preallocation*, the main allocation loop describes our proposed heuristic algorithm, and the resulting incidence matrix is the final proposed batch allocation. See main text for details.

There are three main steps to our heuristic algorithm, as shown in Figure 2: (i) *preallocation*, (ii) *stochastic batch allocation*, and (iii) *combining the results*. The input to the heuristic are the two vectors $\boldsymbol{t}$ and $\boldsymbol{b}$, where $\boldsymbol{t}$ is the number of subjects in each treatment group, and $\boldsymbol{b}$ is the number of subjects that will be allocated to each batch. The details are indicated in the flow chart in Figure 3.

The first step is to perform *preallocation* (Figure 2A), by updating $\boldsymbol{t}$ to $\boldsymbol{t}^{\boldsymbol *}$, as $\boldsymbol{t} = B \boldsymbol{q} + \boldsymbol{t}^{\boldsymbol *}$, with $B$ the number of batches, all $i \in {1, \ldots, T}: 0 \leq \boldsymbol{t}^{\boldsymbol *}_i < B$, and $\boldsymbol{q}$ the vector containing, for each treatment group, the number of subjects allocated to each batch. Similarly, $\boldsymbol{b}$ is updated to $\boldsymbol{b}^{\boldsymbol *}$, as $\boldsymbol{b}^{\boldsymbol *} = \boldsymbol{b} - \boldsymbol{q}$. The initial incidence matrix $\boldsymbol{N^*}$ is then obtained as $\boldsymbol{1}_B \boldsymbol{q}^T$, and $\boldsymbol{\Lambda}$ and $\boldsymbol{N}$ are initialized as $\boldsymbol{0}_{T \times T}$ and $\boldsymbol{0}_{B \times T}$, respectively.

In the second step, the remaining subjects are allocated one batch at a time, for $k = 1, \dots, B$. As each treatment now has fewer subjects remaining than there are batches, this is a binary allocation problem where each treatment is allocated at most once per batch. The treatments that can still be chosen are kept in the set S, while the chosen treatments in a batch are iteratively added to the set C (and removed from S). First treatments where $\boldsymbol{t}^{\boldsymbol *}_i = B - (k - 1)$ and those that have not yet been chosen (which have a zero on the diagonal of the concurrence matrix) are allocated. In case no treatments have been selected yet, a pair of treatments is chosen with the lowest value in $\boldsymbol{\Lambda}$. Subsequent treatments are chosen one at a time by taking the treatment with the lowest column-sum in $\boldsymbol{\Lambda}_{\textrm{C},\textrm{S}}$. The incidence and concurrence matrices are updated every round, after choosing all treatments for a batch. When all batches have been allocated, the final incidence matrix is the sum of $\boldsymbol{N^*}$ and $\boldsymbol{N}$. As this is a heuristic for structured stochastic batch allocation, we call it SBA.

### 2.4. Performance evaluation

As the proposed algorithm is a heuristic, it cannot guarantee an optimal allocation. Ideally, one would therefore calculate the optimal allocation and compare it to the allocations from the proposed heuristic. Unfortunately, this is only possible for small and/or balanced experimental settings. SBA was therefore instead compared to a random allocation strategy where the preallocation is done as specified above followed by a random binary allocation (RBA). Note that a completely random allocation of cohort to batches can lead to allocations with confounding and was found to perform worse than either of the two algorithms considered here, the results are therefore not shown.

The comparison of the allocation procedures was done by comparing the D-criterium score of the allocation for a standard model including intercept, treatment, and batch. D-criterium is here calculated as the determinant of the information matrix $\boldsymbol{X}^T\boldsymbol{X}$, with $\boldsymbol{X}$ the design matrix. The determinant of the inverse of the information matrix is the generalized variance. Minimizing that determinant is equivalent to maximizing the determinant of the information matrix (Goos, 2002; Pukelsheim, 2006). The design matrix consists of subjects in rows and variables in the columns. Categorical variables are recoded into dummy/indicator variables, and the indicators for batches that are suggested by our algorithm are part of this matrix. This makes the ranking of the allocations possible, without needing further calculations to get the proper score. When the D-criterium is zero, there is confounding and not all aspects of the model can be estimated. When it is different from zero, higher numbers indicate better designs for a specific setting, but the values for different settings are not directly comparable.

The search space is estimated by $\prod{T \choose {b_k}}$ after preallocation. This overestimates the actual number of calculations needed but serves well as a proxy for the actual search space.

### 2.5. Evaluated settings

In order to evaluate our approach with regards to handling settings that differ in terms of balance and sample size, and thus different aspects of the allocation, four example settings were chosen (Table 1), none of which can be simplified by the preallocation step. A note on how confounding can occur in these settings can be found in Section 4.

**Table 1.** The four settings considered for the allocation simulations. The search space is estimated by $\prod{T \choose {b_k}}$. Timings are median times in seconds required to complete one run of 1000 allocations, calculated over 1000 runs

Setting	Sample sizes per group (the vector $\boldsymbol{t}$) size	Max batch space	Search RBA	Median time (s) SBA	
A	6, 6, 6, 6, and 6	3	$10^9$	0.02	0.08
B	10, 10, 10, 10, 10, 10, 10, 10, 10, and 10	5	$10^{45}$	0.55	0.60
C	6, 7, 8, 8, and 9	3	$10^{12}$	0.02	0.12
D	5, 5, 8, 8, 10, 10, 12, 12, 15, and 15	5	$10^{45}$	9.38	0.55

**Fig. 4.**
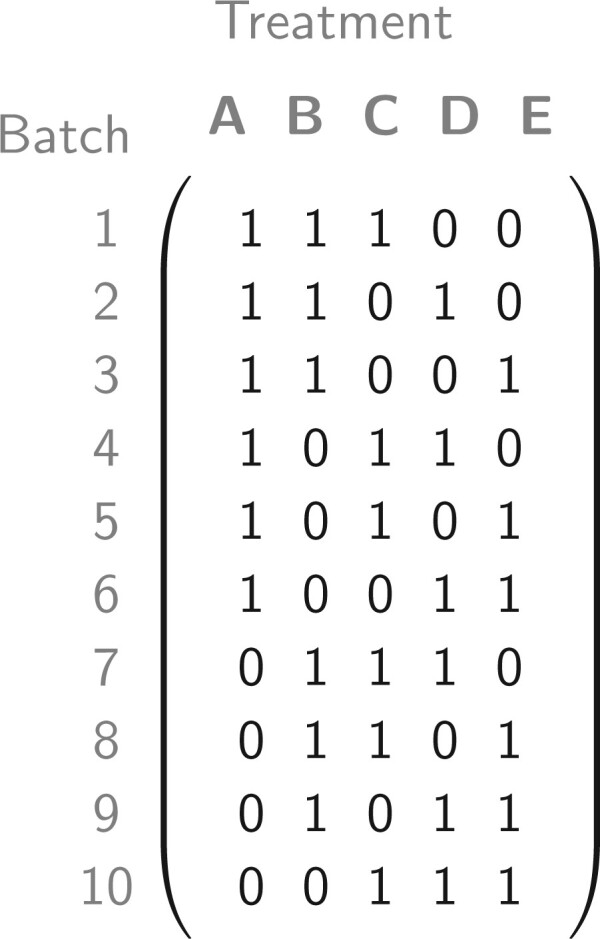
The optimal batch allocation (with ordered batches) for a standard balanced incomplete-block setting with six replicates of five treatments and a batch size of three. Here, each batch contains a single block, as in block randomization.

*Setting A*, was chosen to evaluate how efficiently SBA can find an optimal allocation where one can easily be found by hand, and consists of 5 treatment groups with 6 subjects, each divided over 10 batches of size 3. The optimal allocation for this setting is presented in Figure 4, where every possible binary batch allocation occurs exactly once and corresponds to a balanced incomplete-block design. The algorithm does not have a separate check to see whether there is a simple solution for a balanced incomplete-block design as presented here, so while the given setting is trivially solved by hand, the algorithm handles it in a generic fashion.

*Setting B* is a larger incomplete-block design with equal treatment group sizes, consisting of 10 treatment groups of 10 subjects each and a maximum batch size of 5. A balanced incomplete-block design analogous to the previous setting would require 252 batches and 126 subjects per group. Instead, here, each group has 10 subjects, which does not allow for a balanced design. The calculation of the highest achievable D-criterium score could take several months due to the very high number of possible allocations and was therefore not calculated. This setting tests a balanced setting, which is more challenging to do by hand.

*Setting C* was designed to test a smaller imbalanced setting, an incomplete-block design with unequal treatment sizes. As in *Setting A*, there are five groups and a maximum batch size of three, but the treatment sizes are 6, 7, 8, 8, and 9. No preallocation is performed as the number of batches is larger than the largest treatment group. This setting tests whether SBA can get stuck in sub-optimal allocations due to the different treatment sizes. Again, the search space precludes calculating the optimal allocation, hence the only comparison that can be made is with the random allocation strategy.

*Setting D* was designed like *Setting B*, to test a larger incomplete-block design with unequal treatment sizes, but here subjects are reallocated such that the treatments now consist of two groups each of sizes 5, 8, 10, 12, and 15. Again, no preallocation is performed, as the number of batches is larger than the largest treatment group. Similar to *Setting C*, this tests whether SBA can get stuck in suboptimal allocations due to the different treatment sizes, but here the differences in treatment sizes are larger.

### 2.6. Code availability

All of the code required to perform allocations for an experimental setting with a single nominal treatment variable, and to confirm the results presented here, is implemented in Julia 1.5.3 https://julialang.org and freely available, including additional documentation, at https://github.com/barsnes-group/sba.

## 3. Results

To validate the output of our heuristic algorithm (SBA), four settings were considered (Table 1): (A) a balanced incomplete-block design with a cohort consisting of 30 subjects equally spread over five treatment groups; (B) an equireplicate incomplete-block design with a cohort consisting of ten treatment groups with ten subjects each; (C) incomplete-block design with unequal treatment sizes; and (D) a larger incomplete-block design with unequal treatment sizes. For details on how the four example settings were selected, please see the Methods section.

To illustrate the performance of SBA vs. a random binary allocation (RBA), 1000 allocations were calculated for each of the settings. To show the variability of the results, 1000 runs, where each run picks the best out of 1000 allocations based on the D-criterium, were performed for each of the settings. Note that across all of the runs, neither RBA, nor SBA, produced a confounding allocation for any of the settings.

**Fig. 5.**
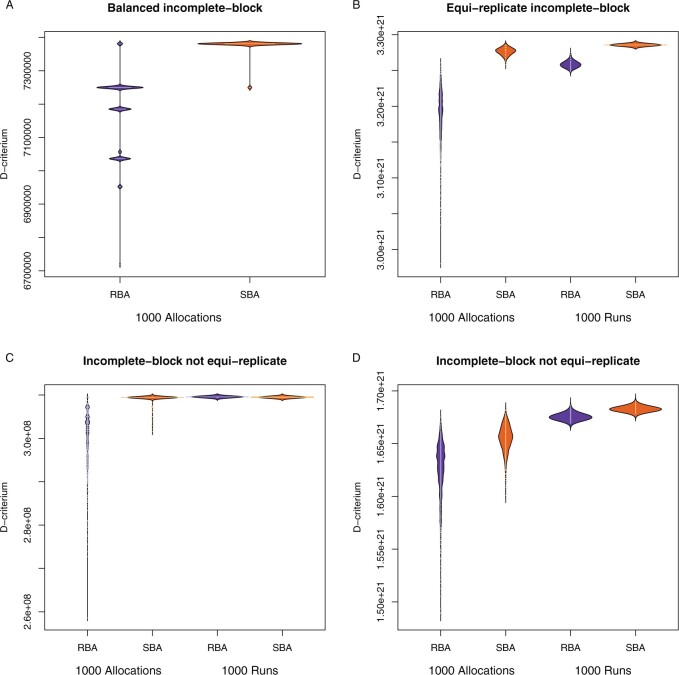
Bean plots of D-criterium distributions. 1000 allocations form a single run and show the efficiency of the algorithms. 1000 runs show the variability of the outcome of runs. Settings follow the numbering as in Table 1.

For *Setting A*, the optimal allocation, given by the incidence matrix in Figure 5, has a D-criterium score of 7 381 125. In a single run, the optimal allocation was produced in 5$\%$ and 96.4$\%$ of the single runs for RBA and SBA, respectively (Figure 5A). Thus, while both algorithms can produce an optimal allocation, SBA does so more often. Across the 1000 iterations of 1000 runs, both algorithms always reached the maximum score, though SBA converged faster on average.

For *Setting B*, all but one of the allocations produced by the random algorithm had a lower D-criterium score than the allocation with the lowest D-criterium score produced by SBA (Figure 5B). Even though, in a single run RBA may have returned a better allocation by chance, SBA thus clearly outperformed RBA in this larger example.

For *Setting C*, both RBA and SBA reached a D-criterium score of 309 561 102 for their best allocation (Figure 5C). As in *Setting A*, however, for a single run, the allocations from SBA presented consistently higher D-criterium scores than RBA. Across the runs, RBA and SBA yielded two different D-criterium scores. The higher score was achieved in 37.5$\%$ and 99.6$\%$ of the runs for SBA and RBA, respectively. However, a closer look at the variances for the simple contrasts revealed that there was only a small difference between the sets of allocations for these two D-criterium scores. For any simple contrast, the difference in variance is less than 7$\%$, thus in practice, the differences between the allocations are minimal.

*Setting D* was chosen to evaluate the performance when both the search space and the imbalance in sample sizes are large. In a single run, the distribution of D-criterium scores was more concentrated toward higher values for SBA compared to RBA. Similarly, in the combined runs, only 2$\%$ of the RBA runs returned an allocation with a D-criterium score at least as high as the median D-criterium score for SBA. In this setting, SBA thus outperformed RBA, though much less clearly compared to *Setting B* (Figure 5D).

**Fig. 6.**
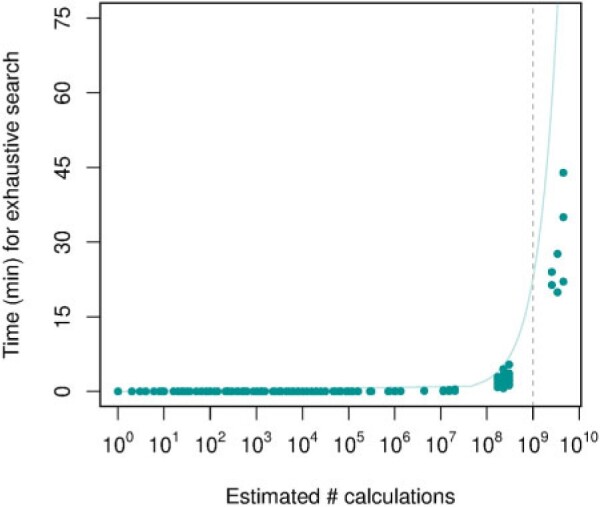
The time needed to calculate optimal allocation vs. the estimated number of calculations required. The vertical dashed line indicates where finding the optimal allocation quickly starts to take a significant amount of time. As an example, the topmost data point represents the setting with six treatment groups, three with five subjects and three with six subjects, and maximum four subjects per batch.

For *Setting A,* it is easy to calculate the optimal allocation and there is no need to run the stochastic algorithm, while for the other settings exhaustive investigation of the search space becomes impractical. When the number of calculations to perform is larger than $10^9$ the time it takes to exhaustively find the best allocation increases dramatically (Figure 6). Our heuristic, however, runs in under a second for all of the considered settings (Table 1).

## 4. Discussion

The results indicate that our heuristic algorithm (SBA) finds better allocations on average compared to an RBA, although by chance this may not be the case for one specific run. In general, neither algorithm exhaustively outperforms the other. For the smaller settings, SBA proved more efficient in finding the best allocations than a random approach, but in this case, computing the optimal allocation might be affordable. As the search space grows, finding the optimal allocation becomes computationally challenging. Then, SBA performs better than RBA, both in speed and quality of the allocations. When a naive search space calculation requires evaluating more than $10^9$ options, it takes a substantial amount of time to find the optimal allocation. For these settings, the recommended procedure is to run SBA until there has been no improvement in the D-criterium score for $x$ runs, where $x$ should be a sufficiently large number chosen as a trade-off between the time spent searching and the possibility of finding an improved allocation.

Note that while the incidence matrix obtained from SBA will always provide a suggested set of batches, the order of the allocations, and which subjects from the given treatments to allocate to which batch, is not defined. The next steps are thus to (i) randomize the order of the batches (premultiply the incidence matrix with a random permutation matrix), (ii) randomly assign subjects to available slots for their treatment, and (iii) randomize the order of the samples for each batch separately to define the processing order.

The evaluated settings were chosen to represent the different aspects relevant to the allocation problem: (i) the level of imbalance in the treatment sizes, (ii) the number of treatments relative to the number of places in each batch, and (iii) the overall size of the problem in terms of the total number of possible allocations. Within each setting, an example was chosen to represent that setting. Note that, due to the preallocation, each example will give the same results for settings where $N^*_i = N_i + B a_i$, and $b^*_k = b_k + \sum a_i$, with $N^*_i$ the new treatment sizes, and $b^*_k$ the new batch sizes.

Note that for each of the settings it is easy to produce batch allocations with confounding by hand, for example, filling the batches one by one, starting by allocating all subjects receiving one treatment first, then all subjects receiving the second treatment, etc. In Setting A, this would lead to all subjects receiving Treatment A being allocated to Batches 1 and 2, all subjects receiving Treatment B being allocated to Batches 3 and 4, and so on. This way no two treatments share a batch, and thus batch and treatment are completely confounded. More subtle would be an allocation that leads to, for example, Treatments A and B sharing a batch more often than Treatments A and C. Any difference between Treatments A and B can then be estimated more precisely than that between Treatments A and C.

By using the D-criterium to compare different allocations of the same set of samples, we treat each comparison between treatments as equally important. In settings where it is not possible to estimate all comparisons between treatments with the same precision, the allocation proposed by the heuristic should be near optimal, so it can either be used directly or as a starting point.

The reason why SBA might pick allocations that are suboptimal in terms of the D-criterium in settings with large sample size imbalance seems inherent in the way the algorithm works: by only keeping track of what has been allocated, but not what is still to be allocated, it consistently tries to allocate the same number of subjects for each treatment. However, when the sample sizes are very different, this may not be an optimal strategy in terms of the overall variance. For the simple contrasts, the difference for the settings we analyzed are minimal and the differences roughly cancel each other out. In such cases, which is better therefore mainly depends on personal preference.

The precision with which we can estimate model parameters is dependent on correlations between the model parameters themselves and the outcome (Kutner *and others*, 2005). In many settings, particularly in the omics field, this is unknown, without trusted proxies. One option is to calculate over a range of (standardized) values, but this can quickly become difficult to interpret. For this reason, we use an approximation of the D-criterium, by calculating the determinant of the information matrix, as a proxy for the inverse of the generalized variance (Pukelsheim, 2006). This is both reasonably fast to calculate and serves as a way to order the obtained batch allocations by generalized variance.

When having larger cohort sizes, it becomes affordable to study multiple variables of interest. It is unfortunately not trivial to extend the suggested heuristic to multiple treatment variables. Take for example, the challenges for the case with two treatment variables ($\beta$ and $\gamma$), without interaction. A first intuition would be to treat this in the same manner as the case of two treatment variables and an interaction, where one may be able to block on the interaction. Note however that by blocking on the interaction, all levels of the interaction will be put together with equal priority. Thus, especially with smaller batches, this can easily lead to suboptimal allocations.

Another multivariable option would be to use multiple concurrence matrices. At each step, one would then first find the minimal column-sums for $\beta$, followed by an investigation of the concurrence matrix for $\gamma$. Note that the chosen treatment levels of $\beta$ will dictate which options are available for $\gamma$. We have not yet investigated whether such a sequential prioritizing will lead to desirable results.

To conclude, where an optimal allocation can be calculated, this is naturally the preferred option. Unfortunately, even for medium-sized experiments, especially with imbalanced cohorts, this quickly becomes intractable. For such settings, we provide a heuristic algorithm to automate the generation of batches for a fixed cohort. By running the heuristic many times and choosing the best overall allocation, a batch allocation that can be expected to be better than a random allocation can quickly be generated. The suggested algorithm thereby greatly simplifies the generation of batch allocations for large studies, especially with imbalanced cohorts and incomplete-block designs, thus making experiments both more reproducible and easier to analyze.
